# Reliability, validity, and sensitivity of the Japanese version of the University of California Los Angeles scleroderma clinical trial consortium gastrointestinal tract instrument: Application to efficacy assessment of intravenous immunoglobulin administration

**DOI:** 10.1111/1346-8138.17202

**Published:** 2024-04-01

**Authors:** Kazuki M. Matsuda, Eiki Sugimoto, Yoshiaki Ako, Marie Kitamura, Mai Miyahara, Hirohito Kotani, Yuta Norimatsu, Teruyoshi Hisamoto, Ai Kuzumi, Takemichi Fukasawa, Shinichi Sato, Ayumi Yoshizaki

**Affiliations:** ^1^ Department of Dermatology, Graduate School of Medicine The University of Tokyo Tokyo Japan; ^2^ Department of Clinical Cannabinoid Research, Graduate School of Medicine The University of Tokyo Tokyo Japan

**Keywords:** autoantibody, cytokine, gastrointestinal symptom, intravenous immunoglobulin, patient‐reported outcome, systemic sclerosis

## Abstract

This study aimed to develop and assess the reliability, validity, and sensitivity of the Japanese version of the University of California Los Angeles Scleroderma Clinical Trial Consortium gastrointestinal tract (GIT) Instrument 2.0 (the GIT score), as an evaluation tool for GIT symptoms in systemic sclerosis (SSc). The Japanese version of the GIT score was constructed using the forward‐backward method. The reliability and validity of this instrument were evaluated in a cohort of 38 SSc patients. Correlation analysis was conducted to assess the relationship between the GIT score and existing patient‐reported outcome measures. Additionally, the sensitivity of the GIT score was examined by comparing GIT scores before and after intravenous immunoglobulin (IVIG) administration in 10 SSc‐myositis overlap patients, as IVIG has recently demonstrated effectiveness in alleviating GIT symptoms of SSc. As a result, the Japanese version of the GIT score exhibited internal consistency and a significant association with the Frequency Scale for the Symptoms of Gastroesophageal Reflux Disease. Furthermore, the total GIT score, as well as the reflux and distention/bloating subscales, displayed moderate correlations with the EuroQol 5 dimensions (EQ‐5D) pain/discomfort subscale and the Short Form‐36 body pain subscale. Notably, following IVIG treatment, there was a statistically significant reduction in the total GIT score and multiple subscales. We first validated the Japanese version of the GIT score in Japanese SSc patients in real‐world clinical settings. This instrument holds promise for application in future clinical trials involving this patient population.

## 1 INTRODUCTION

Systemic sclerosis (SSc) is a complex connective tissue disease typified by widespread inflammation, vasculopathy, and severe fibrosis affecting various organs, including the skin, lungs, and gastrointestinal tract (GIT).^1^ The fibrotic process particularly compromises the GIT by inducing hypomotility, leading to a spectrum of manifestations throughout both the upper and lower GIT, such as gastroesophageal reflux disease (GERD) and intestinal pseudo‐obstruction. Notably, as many as 90% of SSc patients suffer GIT abnormalities, which significantly associates with a marked decline in health‐related quality of life (HRQOL),^2^ extended duration of hospitalization, and, in severe cases, increased mortality rates.^3^

Although the current therapeutic modalities for addressing the GIT involvement of SSc have been constrained, the emergence of innovative treatment approaches with disease‐modifying potential, including biologics^4^, ^5^ and autologous hematopoietic stem cell transplantation,^6^ harbors promise for more efficacious outcomes. Furthermore, recent investigations have underscored the advantages of intravenous immunoglobulin (IVIG), one of the conventional agents being tried for SSc management characterized by its low adverse event profile, for mitigating the GIT symptoms of SSc.^7^ As such, the development of clinical outcome measures that are robust, valid, and sufficiently sensitive for use in clinical trials is imperative to assess the efficacy of these groundbreaking therapies on the GIT symptoms of SSc.

The recent trend towards the integration of solid methodologies for capturing patient perspectives in clinical trials has assumed escalating importance in regulatory decision‐making, aiming to enhance the ‘patient‐centeredness’ of drug development processes. Consequently, patient‐reported outcome measures (PROMs) have become increasingly pertinent in the context of SSc. Given the substantial heterogeneity and complexity of the clinical manifestations in SSc patients, their evaluation necessitates a multidimensional approach. For instance, certain patients may exhibit severe symptoms related to upper GIT involvement, such as reflux, while others may predominantly present complaints attributed to lower GIT abnormalities, such as distention and bloating. Furthermore, conditions that appear mutually exclusive, such as diarrhea and constipation, may paradoxically coexist at varying times throughout the disease progression. This co‐occurrence further complicates the comprehensive communication of the full spectrum of GIT symptoms between patients and clinicians.

In an effort to develop a PROM to subjectively and holistically quantify GIT involvement in SSc patients, Khanna et al. conceptualized the SSc‐GIT 1.0 in 2007.^8^ This tool was initially formulated as a 52‐item questionnaire, the content of which was guided by an extensive literature review, expert consensus, and the findings from two focus groups. Subsequently, in 2009, Khanna et al. introduced a more concise and refined version known as the University of California Los Angeles (UCLA) Scleroderma Clinical Trial Consortium (SCTC) GIT 2.0 Instrument (the GIT score), which comprises 34 items.^9^ Evidence suggests that the GIT score exhibits commendable test–retest reliability. Furthermore, both the total and subscale scores were shown to effectively differentiate between patients with mild, moderate, and severe self‐rated GIT involvement. Thus, its application in both clinical trials and routine patient care has been strongly endorsed.

The GIT score has been adapted and validated in multiple languages, including but not limited to French,^10^ Dutch,^11^ Italian,^12^ Romanian,^13^ and Chinese.^14^ Although a Japanese translation of the questionnaire has been made available by Khanna et al. online, it has yet to undergo validation within the Japanese population. In light of this, we undertook the reformation of the Japanese version of the GIT score, based on its original counterpart. This newly adapted tool was then implemented in a cohort of Japanese patients with SSc in our clinic, and its reliability and validity were evaluated using statistical methodologies. We also assessed the correlation between the GIT scores and clinical manifestations or autoantibody profiles of SSc patients in Japan. Furthermore, we gauged the sensitivity of Japanese version of the GIT score by comparing scores before and after the administration of IVIG, which demonstrated rapid improvement of GIT symptoms of SSc in a previous study.^7^ In this study, our primary objective was to establish this questionnaire as a benchmark tool for evaluating therapeutic efficacy in clinical trials involving the Japanese population.

## 2 MATERIALS AND METHODS

### 2.1 Translation

We utilized the “forward‐backward method”^15^ to construct a Japanese adaptation of the GIT score. The process began with independent translations by two translators (K.M.M. and E.S.), both native speakers of Japanese. They then came together to scrutinize each item, identifying and resolving any potential points of confusion or ambiguity until they reached a consensus (Data S1). This intermediate version of the instrument was then tested on 10 non‐bilingual SSc subjects with no issues arising in relation to clarity or comprehension. Subsequently, this version underwent back‐translation by two bilingual translators. The English rendition produced from this process was critically reviewed by two native English speakers, who found no need for further modifications (Data S2).

### 2.2 Patients

We consecutively recruited Japanese patients with SSc visiting our scleroderma center outpatient clinic from November 2022 until April 2023 to assess the reliability and validity of Japanese version of the GIT score. All the SSc patients fulfilled the classification criteria established by the American College of Rheumatology and European League Against Rheumatism in 2013.^16^ We also sequentially enrolled patients with SSc‐myositis overlap admitted to our wards from April 2023 until August 2023 for IVIG administration for evaluating the sensitivity of the Japanese version of the GIT score. This study was approved by the University of Tokyo Ethical Committee (Approval Number 0695). Written informed consent was obtained from all the subjects.

### 2.3 Clinical data acquisition

Clinical data were collected by retrospective review of electric medical records. We gathered basic patient information, symptoms, medications, and laboratory findings from the closest time point from the date of the GIT score evaluation. SSc patients were categorized by LeRoy's classification rule into diffuse cutaneous SSc (dcSSc), limited cutaneous SSc, or overlap syndrome.^17^ Skin thickness was semiquantitatively examined by the modified Rodnan total skin thickness score.^18^ Interstitial lung disease (ILD), pulmonary hypertension, and scleroderma renal crisis were diagnosed as previously described.^19^

### 2.4 Autoantibody detection

Autoantibodies in the serum samples were evaluated using an autoantibody array assay (A‐Cube) as previously described.^20^, ^21^ Briefly, a total of 65 antigens of 43 autoantibodies associated with SSc, Sjogren syndrome, primary biliary cholangitis, myositis, and overlap syndrome, with FLAG‐GST‐tag on the N‐terminus were synthesized in vitro with a wheat germ cell‐free translation system,^22^ from human complementary deoxyribonucleic acid library entry clones.^23^ The synthesized proteins were captured on array plates under wet conditions by affinity between the glutathione S‐transferase (GST) tags and glutathione coated over the glass slides.^24^ The slides were consequently treated with serum samples diluted in the blocking buffer and fluorescence‐labeled antihuman immunoglobulin G (IgG) antibody (Ab). After the slides were washed and air‐dried, the plates were scanned by a fluorescence imager (Figure S1a). The negative control spots were prepared using distilled water instead of messenger ribonucleic acid (mRNA) during protein preparation. The positive control spots were prepared using mRNA encoding human IgG for protein synthesis. The autoantibody quantification was performed based on the fluorescent values obtained from reactions of serum with the protein spots. The level of each autoantibody was calculated as below:

$$\text{index value}=\frac{\;F_{\text{autoantigen}}-F_{\text{negative control}}}{F_{\text{positive control}}-F_{\text{negative control}}}\times 100$$

where $F_{\text{autoantigen}}\;\text{is the fluorescent intensity of the autoantigen spot}$, $F_{\text{negative control}}\;\text{is the fluorescence intensity of the negative control spot}$, and $F_{\text{positive control}}\;\text{is the fluorescence intensity of the positive control spot}$.

The cut‐off value of each autoantigen was determined based on the mean + 3 standard deviations (SDs) of healthy controls.

### 2.5 Cytokine measurement

The serum levels of cytokines were measured by Luminex Discovery Assay Human Premixed Multi‐Analyte Kit (R&D Systems, Minneapolis, MN, USA) according to the manufacturer's protocol. The evaluated cytokines were as follows: tumor necrosis factor‐alpha (TNF‐α), interleukin‐6 (IL‐6), IL‐10, IL‐27, vascular endothelial growth factor (VEGF), interferon‐gamma (IFN‐γ), IL‐31, IL‐1‐alpha, IL‐4, IL‐17, B‐cell activating factor (BAFF) belonging to the tumor necrosis factor family, IL‐13, interferon‐alpha (IFN‐α), and IL‐23.

### 2.6 Patient‐reported outcome measures

Patients completed the Japanese version of the GIT score, Medical Outcomes Short Form (SF)‐36,^25^ the EuroQol 5 dimensions (EQ‐5D) with five levels tool,^26^ and the F‐scale.^27^ UCLA SCTC GIT 2.0 comprises 34 items divided into seven domains: reflux, distention/bloating, diarrhea, fecal soilage, constipation, emotional well‐being, and social functioning.^9^ Each domain is rated from 0 (indicating better HRQOL) to 3 (representing poorer HRQOL), with the exception of the diarrhea and constipation domains, which have ranges of 0–2 and 0–2.5, respectively. The overall GIT score is the mean score of six out of the seven domains, excluding constipation. The original version in English is accessible online at http://uclascleroderma.researchcore.org/.

The SF‐36 is a broad‐spectrum measure of health status, comprising 36 items that evaluate eight distinct domains.^25^ Four scales examine physical health, namely physical functioning (10 items), bodily pain (two items), role limitations resulting from physical health perceptions (four items), and overall health perceptions (five items). An additional four scales are dedicated to mental health, which include mental health (five items), role limitations due to emotional concerns (three items), vitality (four items), and social functioning (two items), alongside a single‐item health transition scale. The physical health scales collectively form the Physical Component Summary (PCS), and the mental health scales together make up the Mental Component Summary (MCS). These summarized scores are normalized to the general population in Japan, which is characterized by a mean ± SD score of 50 ± 10.^28^ A standard 4‐week recall period was implemented.

The EQ‐5D questionnaire with five levels is a generic instrument to quantify HRQOL.^26^ The EuroQoL Group developed and tested this tool for the purpose of providing measurable health outcomes. In an initial study with SSc patients, the Italian version of this tool proved to be valid.^12^ The EQ‐5D is composed of two primary components. The first section, known as the EQ‐5D profile, generates a health profile derived from a descriptive system. This system defines health based on five dimensions: “mobility”, “self‐care”, “usual activities”, “pain or discomfort”, and “anxiety or depression.”. Each dimension offers three response categories indicating no problems, some problems, or extreme problems. The second component of the questionnaire is the EQ‐5D Visual Analogue Scale, which evaluates the overall HRQOL on a scale from 0 (the worst possible health state) to 100 (the best possible health state). For this study, a standard 4‐week recall period was employed.

The F‐scale refers to the Frequency Scale for the Symptoms of GERD (FSSG), which is a self‐report questionnaire used to assess the frequency and severity of GERD‐related symptoms, originally developed in Japan.^27^ The FSSG consists of 12 items grouped into two subscales: a reflux‐related subscale (acid regurgitation and heartburn) and a dysmotility‐related subscale (including symptoms such as non‐cardiac chest pain, a sensation of a lump in the throat, belching, etc.). Each item is rated on a four‐point scale (never = 0, occasionally = 1, sometimes = 2, often = 3), and the scores are added together to provide a measure of the severity of GERD symptoms.

### 2.7 Statistical analysis

We analyzed average scores, SDs, ranges, and the percentage of missing data. The floor and ceiling effects of the GIT score were determined by calculating the percentage of participants who scored at the extreme lower (floor) and upper (ceiling) limits. We gauged the internal consistency of the GIT score through Cronbach's *α*.^29^ We evaluated the construct convergent validity by examining the relationship among the GIT score, the EQ‐5D, and the SF‐36 domains, using Spearman's rho to measure correlations. The association between the GIT scores and clinical manifestations or autoantibody profiles was investigated by logistic regression analyses. Data analysis was performed using Stata 15/IC (StataCorp, College Station, TX, USA), GraphPad Prism 9 (GraphPad Software, Boston, MA, USA), R, RStudio, and R packages “dplyr,” “ggplot2,” “hrbrthemes,” “ggcorrplot,” and “ComplexUpset.” We set the threshold for statistical significance at *P* < 0.05.

## 3 RESULTS

### 3.1 Study population

We recruited 38 patients with SSc for the assessment of the reliability and validity of the Japanese UCLA SCTC GIT 2.0 (Table 1). A large majority were female (94%), with mean age of 65 years and SD of 11 years, all of whom were of Japanese ethnicity. The proportion of the patients classified into dcSSc was 21%. None of the subjects within this cohort had been classified as overlap with myositis. Comprehensive autoantibody screening using A‐Cube revealed anti‐centromere Ab, anti‐topoisomerase I Ab, anti‐RNA polymerase III Ab, and anti‐U1‐RNP (U1 ribonucleoprotein) Ab with prevalence rates of 45%, 26%, 16%, and 5%, respectively (Figure S1b,c).

**TABLE 1:** Background of the patients for reliability and validity assessment.

Number of patients	38
Basic demographics	
Age, years	65 ± 11
Female, *n* (%)	36 (95%)
Disease duration, years	9 ± 7
Body mass index	20 ± 3
Autoantibodies	
Anti‐topoisomerase I antibody, *n* (%)	10 (26%)
Anti‐centromere antibody, *n* (%)	17 (45%)
Anti‐RNA polymerase III antibody, *n* (%)	6 (16%)
Anti‐U1 ribonucleoprotein antibody, *n* (%)	2 (5%)
Type of systemic sclerosis	
Limited cutaneous systemic sclerosis, *n* (%)	28 (74%)
Diffuse cutaneous systemic sclerosis, *n* (%)	8 (21%)
Overlap with SLE, *n* (%)	2 (5%)
Overlap with PM/DM, *n* (%)	0 (0%)
Skin manifestations	
Diffuse skin sclerosis, *n* (%)	10 (26%)
Modified Rodnan total skin thickness score	13 ± 9
Puffy fingers, *n* (%)	30 (79%)
Telangiectasia, *n* (%)	20 (53%)
Calcinosis, *n* (%)	5 (13%)
Peripheral angiopathy	
Normal, *n* (%)	4 (11%)
Raynaud's phenomenon, *n* (%)	16 (42%)
Pitting scars, *n* (%)	11 (29%)
Digital ulcers, *n* (%)	6 (16%)
Gangrenes, *n* (%)	1 (3%)
*N* of ulcered fingers, *n* (%)	3 ± 4
Organ involvements	
Interstitial lung disease, *n* (%)	16 (42%)
Pulmonary hypertension, *n* (%)	4 (11%)
Scleroderma renal crisis, *n* (%)	1 (3%)
Medications	
PPI, *n* (%)	34 (89%)
Calcium channel blockers, *n* (%)	7 (18%)
Corticosteroids, *n* (%)	13 (34%)
Endothelin receptor antagonists, *n* (%)	18 (47%)
Objective clinical outcome measures	
%FVC	97 ± 19
%DLco	89 ± 18
KL‐6	245 ± 75
SP‐D	72 ± 36
Serum albumin, g/dL	4.4 ± 1.0
Serum TG, mg/dL	134 ± 55
Patient‐reported outcome measures	
F‐scale	
Reflux score	7 ± 6
Dyspeptic score	6 ± 5
Total score	13 ± 11
SF‐36	
PF	77 ± 22
RP	71 ± 28
BP	59 ± 24
GH	46 ± 15
VT	55 ± 20
SF	77 ± 27
RE	79 ± 26
MH	70 ± 18
Physical Component Summary	42 ± 13
Mental Component Summary	50 ± 8
EQ‐5D	
Mobility	0.39 ± 0.72
Self‐care	0.21 ± 0.47
Usual activities	0.66 ± 0.88
Pan/discomfort	0.87 ± 0.91
Anxiety/depression	0.42 ± 0.76
VAS	66 ± 18

Abbreviations: BP, bodily pain; DLco, Diffusing Capacity of the Lungs for Carbon Monoxide; DM, Dermatomyositis; EQ‐5D stands for EuroQol 5 dimensions; FVC, Forced Vital Capacity; GH, general health; KL‐6, Krebs von den Lungen‐6; MCS, mental component summary; MH, mental health; PCS, physical component summary; PF, physical functioning; PM, Polymyositis; PPI, Proton Pump; Inhibitor; RE, role emotional; RP, role physical; SP‐D, Surfactant Protein D; SF, social functioning; SF‐36, Medical Outcomes Short Form‐36; SLE, Systemic Lupus Erythematosus; TG, Triglycerides; VAS stands for "visual analogue scale; VT, vitality.

*Note*: Unless noted otherwise, values are means ± SD.

### 3.2 Reliability

The average GIT score was 0.32 (SD 0.35), with 24% reporting no symptoms (total score = 0), 50% reporting mild symptoms (total score = 0.01–0.49), 21% reporting moderate symptoms (total score = 0.50–1.00), and 5% reporting severe symptoms (total score = 1.01 or above). Multi‐item subscales displayed a Cronbach's *α* ranging from 0.47 to 0.93 (Table 2). A significant floor effect was evident for the total score and all its subscales, ranging from 24% (total score) to 89% (fecal soilage), while there was no observable ceiling effect.

**TABLE 2:** Descriptive statistics and internal consistency reliability of the Japanese University of California Los Angeles scleroderma clinical trial consortium gastrointestinal tract instrument 2.0.

Subclass	Mean (SD)	Minimum	Maximum	Cronbach's *α*	Floor effect (%)	Ceiling effect (%)
Reflux	0.25 (0.37)	0	1.63	0.77	17 (45%)	0 (0%)
Distention/bloating	0.70 (0.67)	0	2.25	0.76	12 (32%)	0 (0%)
Fecal soilage	0.13 (0.41)	0	2	NA	34 (89%)	0 (0%)
Diarrhea	0.43 (0.55)	0	2	0.47	20 (53%)	0 (0%)
Social functioning	0.21 (0.37)	0	1.5	0.76	24 (63%)	0 (0%)
Emotional well‐being	0.17 (0.33)	0	1.67	0.78	21 (55%)	0 (0%)
Constipation	0.36 (0.53)	0	1.75	0.73	19 (50%)	0 (0%)
Total GIT score	0.32 (0.35)	0	1.28	0.93	9 (24%)	0 (0%)

Abbreviations: GIT, gastrointestinal tract; NA, not applicable; SD, standard deviation.

### 3.3 Validity

The reflux subscale and the distention/bloating subscale of the GIT score showed strong and significant correlation with the total score, the reflux subscale, and the dyspepsia subscale of the F‐scale (Table 3). The total GIT score and the reflux and distention/bloating subscales also demonstrated moderate correlations with the EQ‐5D pain/discomfort subscale and the SF‐36 BP subscale. Furthermore, there was a statistically significant, although weak, correlation between selected GIT subscales and certain SF‐36 domains and components.

**TABLE 3:** Spearman's correlation coefficients among patient‐reported outcome measures.

	Reflux	Distention/bloating	Fecal soilage	Diarrhea	Social functioning	Emotional well being	Constipation	Total
F‐scale								
Reflux	**0.79** ****	**0.71** ****	0.10	**0.51** **	**0.43** **	**0.47** **	0.21	**0.71** ****
Dyspepsia	**0.58** ***	**0.77** ****	0.18	**0.60** ***	**0.48** **	**0.45** **	**0.34** *	**0.75** ****
Total	**0.72** ****	**0.77** ****	0.15	**0.57** ***	**0.47** **	**0.48** **	0.29	**0.77** ****
EQ‐5D								
Mobility	0.27	0.10	−0.05	−0.14	0.05	−0.07	−0.02	0.05
Self‐care	0.24	0.21	−0.16	0.05	−0.07	0.02	0.11	0.14
Usual activities	**0.44** **	0.28	−0.03	0.12	0.09	0.12	0.20	0.25
Pain/discomfort	**0.38** *	**0.47** **	0.03	0.09	0.30	0.23	**0.33** *	**0.37** *
Anxiety/depression	0.08	0.13	−0.02	0.10	0.03	0.13	0.12	0.12
VAS	−0.18	−0.19	0.03	0.04	−0.06	−0.13	−0.11	−0.13
SD‐36								
PF	**−0.39** *	−0.12	0.10	0.13	−0.06	0.09	0.08	−0.05
RP	**−0.36** *	−0.18	−0.02	−0.15	−0.21	0.05	−0.18	−0.20
BP	**−0.49** **	**−0.49** **	−0.09	−0.26	**−0.33** *	**−0.40** *	−0.25	**−0.47** **
GH	−0.21	**−0.36** *	−0.13	−0.10	−0.08	**−0.37** *	−0.08	−0.30
VT	**−0.34** *	−0.23	0.04	−0.06	−0.09	−0.03	0.03	−0.18
SF	−0.27	−0.12	0.18	−0.16	0.08	−0.07	0.12	−0.12
RE	−0.32	−0.28	0.01	−0.05	0.00	−0.04	0.13	−0.18
MH	−0.01	0.03	0.12	−0.05	0.23	0.01	0.20	0.06
PCS	**−0.51** **	**−0.34** *	−0.12	−0.00	−0.23	−0.09	−0.13	−0.31
MCS	−0.03	−0.10	0.07	−0.01	0.08	−0.16	0.12	−0.03

*Note*: Boldface letters indicate values that are statistically significant.

Abbreviations: BP, bodily pain; EQ‐5D, EuroQol 5 dimensions; GH, general health; MCS, mental component summary; MH, mental health; PCS, physical component summary; PF, physical functioning; RE, role emotional; RP, role physical; SF, social functioning; SF‐36, Medical Outcomes Short Form‐36; VAS, visual analogue scale; VT, vitality.

* *P* < 0.05.

** *P* < 0.01.

*** *P* < 0.001.

**** *P* < 0.0001.

### 3.4 Association between clinical features

**FIGURE 1:**
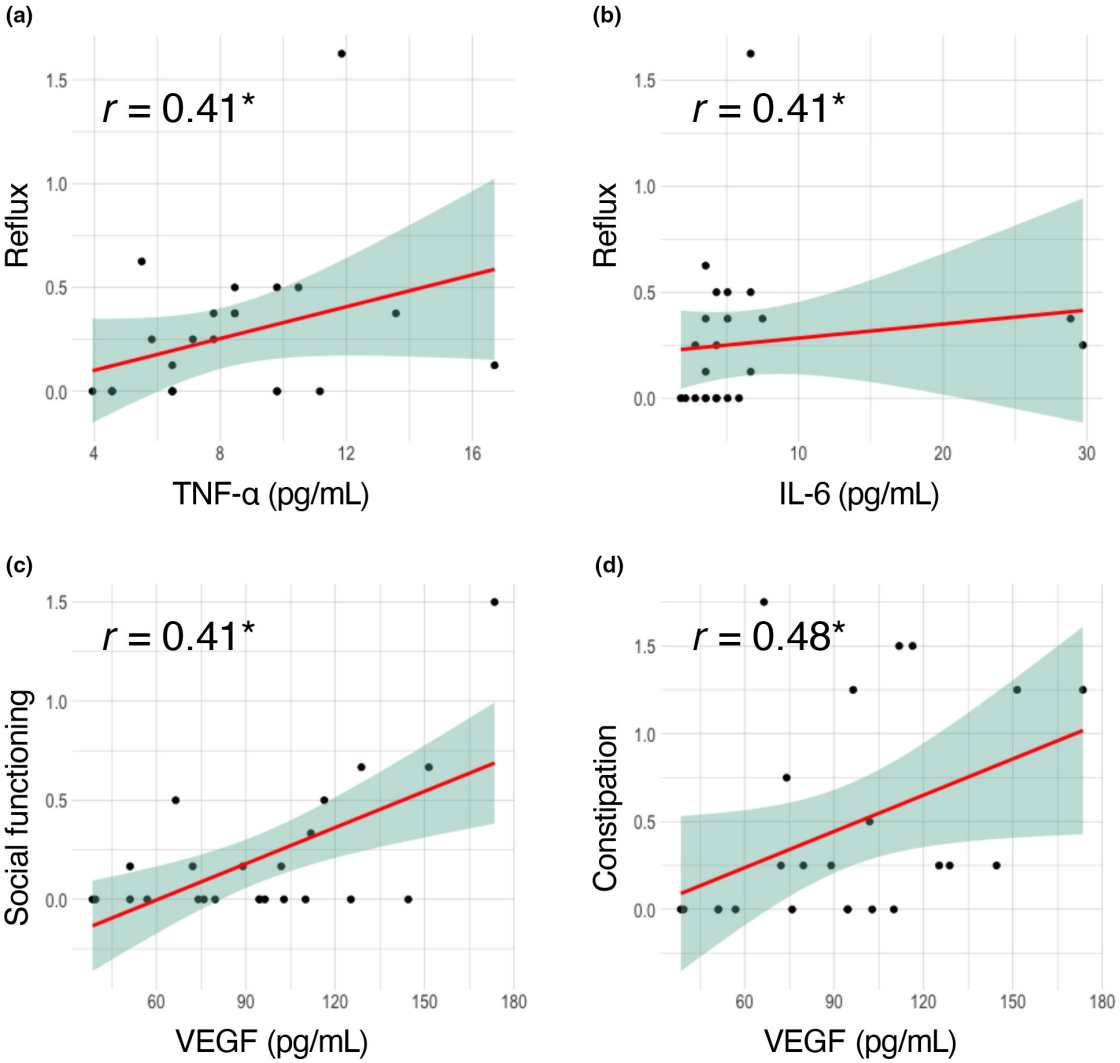
Correlation between serum cytokine levels and Japanese University of California Los Angeles scleroderma clinical trial consortium gastrointestinal tract instrument 2.0 scores. Scatter plots of tumor necrosis factor‐alpha (TNF‐α) versus reflux subscale (a), interleukin‐6 (IL‐6) versus reflux subscale (b), vascular endothelial growth factor (VEGF) versus social functioning subscale (c), and VEGF versus constipation subscale (d). *r*: Spearman's rho. *P < 0.05. The red line and the green area represent the regression line and its 95% confidence interval, respectively.

No statistically significant correlation was observed between the GIT scores and the clinical manifestations of SSc, as indicated in Table S1. Similarly, there was no significant association between the GIT scores and Ab profiles, as detailed in Table S2. Meanwhile, a statistically significant correlation was observed between the serum levels of several cytokines and specific GIT subscales, as outlined in Table 4. Notably, there was a significant correlation between serum levels of TNF‐α or IL‐6 and the reflux subscale, as illustrated in Figure 1a,b. Additionally, a significant correlation was found between serum levels of VEGF and the social functioning or constipation subscales, as depicted in Figure 1c,d.

**TABLE 4:** Association between serum cytokine levels and University of California Los Angeles scleroderma clinical trial consortium gastrointestinal tract instrument scores.

Cytokine	Serum level (pg/mL)	Spearman's rho							
		Reflux	Distention/bloating	Fecal soilage	Diarrhea	Social functioning	Emotional well‐being	Constipation	Total GIT score
TNF‐α	8.2 ± 3.1	**0.41** *	−0.05	−0.26	−0.10	−0.10	−0.11	−0.11	−0.05
IL‐6	6.5 ± 7.2	**0.41** *	−0.06	−0.35	−0.06	−0.23	−0.12	0.12	−0.04
IL‐10	4.2 ± 2.2	−0.09	−0.23	−0.18	−0.31	−0.17	−0.21	−0.18	−0.27
IL‐27	196 ± 68	−0.34	−0.13	0.04	−0.25	0.18	0.07	0.06	−0.11
VEGF	94 ± 35	0.01	−0.01	−0.02	0.12	**0.41** *	0.12	**0.48** *	0.10
IFN‐γ	36 ± 19	0.12	−0.18	−0.15	−0.16	−0.26	−0.17	−0.24	−0.20
IL‐31	62 ± 31	−0.15	−0.06	0.11	0.03	−0.26	0.00	0.00	−0.07
IL‐1α	9.4 ± 4.1	0.06	−0.29	−0.26	−0.19	−0.31	−0.16	−0.20	−0.27
IL‐4	102 ± 30	0.06	−0.28	−0.19	−0.17	−0.30	−0.17	0.18	−0.25
IL‐17	4.9 ± 1.3	−0.33	−0.38	−0.14	−0.19	−0.14	−0.25	0.00	−0.35
BAFF	920 ± 269	0.14	−0.17	−0.13	−0.12	−0.22	−0.10	−0.18	−0.15
IL‐13	354 ± 106	0.01	−0.06	−0.02	−0.19	0.21	0.05	0.36	−0.06
IFN‐α	3.3 ± 1.0	0.11	0.04	0.16	0.20	−0.09	0.21	−0.02	0.11
IL‐23	413 ± 166	0.07	−0.29	−0.19	−0.27	−0.18	−0.25	−0.09	−0.26

*Note*: Boldface letters mean statistically significant.

Abbreviations: BAFF, B‐cell activating factor belonging to the tumor necrosis factor family; GIT, gastrointestinal tract; IFN, interferon; IL, interleukin; TNF‐α, tumor necrosis factor‐alpha; VEGF, vascular endothelial growth factor.

* *P* < 0.05.

### 3.5 Sensitivity

**FIGURE 2:**
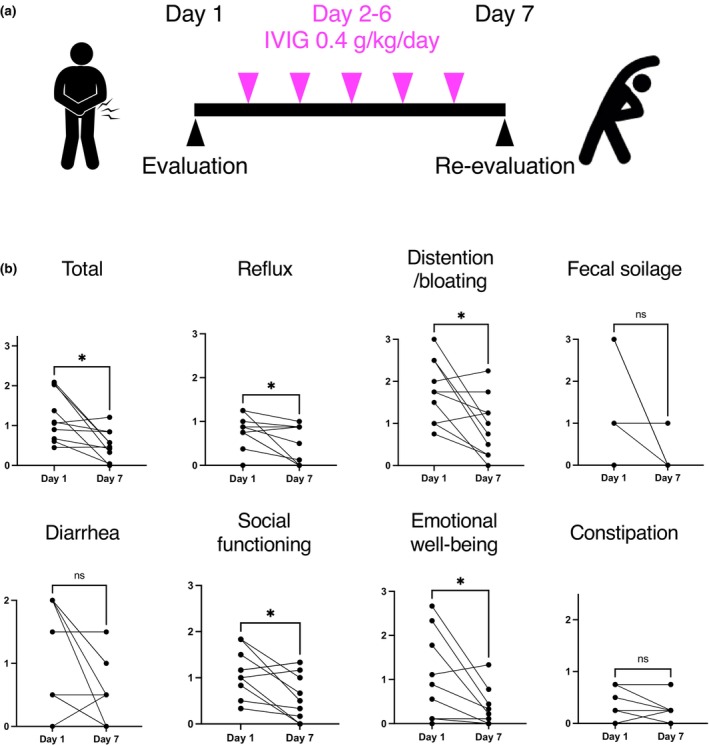
Sensitivity of the University of California Los Angeles scleroderma clinical trial consortium gastrointestinal tract instrument 2.0 in relation to intravenous immunoglobulin (IVIG) administration. (a) Schematic figures of the study design. The Japanese version of the gastrointestinal tract (GIT) score was analyzed on days 1 and 7. IVIG 2 g/kg was administered over 5 days (days 2–6). (b) The total GIT score and subscales before and after IVIG administration. *P* values were calculated by Wilcoxon signed‐rank test. *P < 0.05.

We enrolled a cohort of 10 Japanese patients diagnosed with SSc‐myositis overlap, with a predominance of nine female patients (90%). Their average age was 65 years, with an SD of 8 years. Among the patients, six patients were positive for anti‐centromere Ab, two were positive for anti‐U3‐RNP Ab positivity, and one was positive for anti‐RNA polymerase III Ab. The Japanese version of the GIT score was administered both before and after IVIG treatment (Figure 2a), revealing a reduction in total GIT scores with statistical significance, as well as the subscales, except for fecal soilage, diarrhea, and constipation (Figure 2b).

## 4 DISCUSSION

In the present study, the Japanese version of the UCLA SCTC GIT 2.0 instrument demonstrated commendable internal consistency and good reliability (Table 2), comparable with its original version.^9^ Additionally, the Japanese version of the GIT score exhibited robust divergent validity demonstrated by significant association with the F‐scale (Table 3), suggesting its usefulness as a tool for evaluating GIT symptoms associated with SSc in real clinical settings. GIT symptoms receive less attention than other symptoms of SSc; GIT manifestations are not evaluated in composite measures of the disease such as the American College of Rheumatology Composite Response Index in Systemic Sclerosis.^30^ The absence of significant correlations between GIT score outcomes and other clinical manifestations of SSc affirmed that GIT involvement in SSc stands as an independent factor (Table S1), warranting separate evaluation.

When contrasted with the original study utilizing the English version,^9^ several baseline differences in the study population were observed (Table 1). The Japanese version assessment was conducted on a smaller patient population (*n* = 38 vs 152), the patients were older (mean age = 65 vs 51 years), and our evaluation indicated lower mean scores in all the subscales: reflux (0.25 vs 0.69), distention/bloating (0.70 vs 1.07), fecal soilage (0.13 vs 0.30), diarrhea (0.43 vs 0.56), social functioning (0.21 vs 0.26), emotional well‐being (0.17 vs 0.49), constipation (0.36 vs 0.43), and total GIT score (0.32 vs 0.66). Furthermore, the prevalence of cases exhibiting a floor effect was notably higher in our cohort, whereas the maximum score tended to be higher in the original study. One explanation might be the higher proportion of patients already treated; most of our patients were already on proton pump inhibitors (89%). Alternatively, one could interpret our study as having enrolled individuals with SSc who had comparatively milder disease manifestations and fewer health impairments. This interpretation finds support in our assessment of HRQOL, revealing mean SF‐36 PCS and MCS scores of 41.2 and 50.4, respectively, in contrast to the original study where these scores were 36.7 and 47.1, respectively. Moreover, our study featured a smaller proportion of patients with dcSSc (22% vs 55%), a factor associated with severe gastrointestinal involvement in SSc.^31^

An advantage of this study is the multidimensional immunophenotyping conducted, which encompassed assessments of serum cytokine levels and autoantibody profiles, aligned with the GIT score outcomes. As a result, our research demonstrates a significant correlation between the serum levels of IL‐6, TNF‐α, and VEGF and some subscales of the GIT scores (Figure 1). Compared with healthy individuals, patients with SSc exhibit elevated serum levels of these cytokines.^32^, ^33^ The role of IL‐6 in SSc is particularly well‐documented, with a substantial experimental evidence base^34^ and clinical observations tying serum IL‐6 levels to manifestations such as skin, muscle, and cardiac involvements.^35^, ^36^ This connection is underscored by the US Food and Drug Administration's approval of tocilizumab, an IL‐6 receptor inhibitor, for treating SSc‐related ILD.^37^ Recently, significant elevation of serum IL‐6 levels in SSc patients with GIT involvements diagnosed by clinical manifestations and gastrointestinal endoscopy has been reported.^38^ However, correlations between IL‐6 and GIT symptom severity have not been consistently observed in other studies using barium radiography^35^ or the Scleroderma Assessment Questionnaire.^39^ Furthermore, the effect of tocilizumab on GIT symptoms in SSc is still debated, with some reports of exacerbation during treatment.^40^ On the other hand, the efficacy of TNF‐α inhibitors in SSc treatment lacks definitive evidence from large‐scale, randomized placebo‐controlled trials, although smaller studies suggest potential benefits.^41^ Interestingly, biopsies from patients with GERD indicate that IL‐6 is the primary cytokine produced by esophageal cells, not TNF‐α.^42^ As for VEGF, although its involvement in SSc pathogenesis is supported by clinical association with diffuse skin sclerosis and animal studies,^43^, ^44^ its link to GIT symptoms in SSc has not been established. The intricate relationship between these cytokines and GIT symptoms in SSc remains elusive, likely due to the disease's complex pathophysiology involving multiple factors such as fibrosis, vasculopathy, and immune dysregulation across different organs,^45^ and inconsistency in the methodology for evaluating GIT symptoms in SSc among previous reports.^35^, ^38^, ^39^

The primary highlight of this study lies in its ability to demonstrate the sensitivity of the GIT score through the improvement observed in the GIT score before and after IVIG administration (Figure 2). In a prior publication, we presented evidence of rapid alleviation of subjective symptoms and imaging findings of SSc‐related GIT symptoms such as intestinal pseudo‐obstruction and, moreover, weight recovery and weaning from total parenteral nutrition following regular monthly IVIG treatments in patients with SSc‐myositis overlap.^7^ Our current study reaffirmed the immediate effectiveness of IVIG, as reflected in the improvement of the GIT score. These findings underscore the utility of the GIT score as a tool for evaluating the effectiveness of IVIG in Japanese SSc patients within real‐world clinical settings and, prospectively, in forthcoming clinical trials.

Our study has several limitations. Initially, it is important to note that the sample size in our study was relatively modest. This limitation could potentially explain our inability to detect any associations between autoantibody profiles and the GIT scores (Table S2), even though certain autoantibodies, such as anti‐U3‐RNP Ab,^46^ are recognized for their association with GIT involvement in SSc. Furthermore, it is worth acknowledging that our evaluation of GIT scores followed a retrospective design and was limited to the patients treated with IVIG. We focused on SSc patients receiving IVIG due to its potential for rapid efficacy,^7^ in contrast to other treatments like proton pump inhibitors, whose effectiveness has been demonstrated over more extended periods.^47^, ^48^ The potential for biases cannot be entirely ruled out from this study design, although we made efforts to minimize them by sequentially enrolling cases. Additionally, it was challenging to definitively differentiate the impact of SSc from myositis on GIT symptoms, as our longitudinal assessment was limited to patients with SSc‐myositis overlap. The sensitivity of the Japanese version of the GIT score should be further validated by long‐term prospective observation of a more extensive cohort of SSc patients, both with and without myositis, and encompassing a range of different treatment approaches. Finally, we should emphasize that the purpose of including IVIG was to explore the utility of the Japanese version of the GIT score to measure longitudinal changes of SSc‐related GIT symptoms in a real‐world clinical setting, not to conclusively prove the efficacy of IVIG. To comprehensively address the efficacy and safety of IVIG in managing SSc‐related GIT symptoms, future studies should aim for a more rigorous investigation, ideally in a prospective, multicenter, randomized, and placebo‐controlled setup.

## FUNDING INFORMATION

No specific funding was received from any bodies in the public, commercial, or not‐for‐profit sectors to carry out the work described in this article.

## CONFLICT OF INTEREST STATEMENT

T.F. and A.Y. belong to the Social Cooperation Program, Department of Clinical Cannabinoid Research, supported by the Japan Cosmetic Association and the Japan Federation of Medium and Small Enterprise Organizations. The remaining authors declare that the research was conducted in the absence of any commercial or financial relationships that could be construed as a potential conflict of interest.

## ETHICS STATEMENT

This study was approved by the University of Tokyo Ethical Committee (approval number 0695).

## Supporting information


Data S1.

Data S2.

Figure S1.

Table S1.

Table S2.

## Data Availability

The data analyzed during the current study are available from the corresponding author on reasonable request.
