# Supplementary material for: Reliability, validity, and sensitivity of the Japanese version of the University of California Los Angeles scleroderma clinical trial consortium gastrointestinal tract instrument: Application to efficacy assessment of intravenous immunoglobulin administration
Source: J Dermatol. 2024 Apr 1;51(6):741–51. doi: 10.1111/1346-8138.17202 (PMC11483899; doi:10.1111/1346-8138.17202)
Supplement: Supplementary file 1 — Data S1. [file JDE-51--s001.pdf]

UCLA SCTC GIT 2.0 質問票

以下の質問は過去7日間の消化器（胃腸）の症状と、その症状があなたの生活にどの程度影響したかについてお尋ねするものです。すべての質問に対して、指示されている通りに選択肢の中から回答を選んでください。どのように回答していいかわからない場合は、できる限り近い回答を選択してください。（□の中にチェックを入れてください。）

<u>この1週間</u>で、以下の事柄が起こった頻度（日数）をお答えください。

	なし	1-2日	3-4日	5-7日
固形物を飲み込むことが困難だった	□	□	□	□
胸に不快な鋭い痛みまたは焼けるような感覚があった（胸焼け）	□	□	□	□
苦いまたは酸っぱい液体が胃から口に上がってくるのを感じた（胃酸の逆流）	□	□	□	□
トマトやオレンジのような「酸味のある」食べ物を食べた時に胸焼けがした	□	□	□	□
吐き戻した（食べたものを少量吐いたり、上がってきたりした）	□	□	□	□
上半身を高くしたり、背中を垂直に立てたりして寝た	□	□	□	□
吐き気があった/気持ちが悪かった	□	□	□	□
吐いた	□	□	□	□

この1週間で、以下の事柄が起こった頻度（日数）をお答えください。

	なし	1-2日	3-4日	5-7日
腹満感（お腹にガスや空気が溜まっている感じ）があった	☐	☐	☐	☐
お腹が膨れることがあり、時にはベルトをゆるめたり、ズボンやシャツのボタンを外す必要があった	☐	☐	☐	☐
少しの食事で満腹になった	☐	☐	☐	☐
おならがたくさん出たり、ガスがたまったりした	☐	☐	☐	☐
トイレに行くのが間に合わず、下着を便で汚してしまった	☐	☐	☐	☐
便がゆるかった	☐	☐	☐	☐

この1週間で、便が以下の状態になったことに気付きましたか。

	いいえ	はい
便が水っぽかった	☐	☐

この1週間で、以下の事柄により社会生活が妨げられた頻度をお答えください（友人や親戚を訪問するなど）。

	なし	1-2日	3-4日	5-7日
吐き気	☐	☐	☐	☐
嘔吐	☐	☐	☐	☐
胃痛	☐	☐	☐	☐
下痢	☐	☐	☐	☐
うっかり下着を汚してしまうかもしれないという心配	☐	☐	☐	☐
膨満感	☐	☐	☐	☐

この1週間で、以下の事柄が起こった頻度（日数）をお答えください。

	なし	1-2日	3-4日	5-7日
腸の問題についての心配や不安を感じた	☐	☐	☐	☐
腸の症状が原因で恥ずかしさを感じた	☐	☐	☐	☐
腸の症状が原因で性生活上の問題が生じ、パートナーとの関係にも問題が生じた	☐	☐	☐	☐
トイレを見つけられないかもしれないという不安があった	☐	☐	☐	☐
腸の症状が原因で精神的に落ち込んだり、弱気になったりした	☐	☐	☐	☐
腸の症状が原因で旅行を避けたり、延期したりした	☐	☐	☐	☐
腸の症状が原因で怒りや苛立ちを感じた	☐	☐	☐	☐
腸の症状が原因で睡眠を妨げられた	☐	☐	☐	☐
「ストレス」や気持ちの乱れにより腸の症状が悪化すると感じた	☐	☐	☐	☐

<u>この1週間</u>で、便が以下の状態になったことに気付きましたか。

	いいえ	はい
硬くなってきた	☐	☐

<u>この1週間</u>で、以下の事柄が起こった頻度をお答えください。

	なし	1-2日	3-4日	5-7日
便秘になったり、排便しきれなかったりした	☐	☐	☐	☐
便が硬かった	☐	☐	☐	☐
排便時に痛みがあった	☐	☐	☐	☐
