# Supplementary material for: Reliability, validity, and sensitivity of the Japanese version of the University of California Los Angeles scleroderma clinical trial consortium gastrointestinal tract instrument: Application to efficacy assessment of intravenous immunoglobulin administration
Source: J Dermatol. 2024 Apr 1;51(6):741–51. doi: 10.1111/1346-8138.17202 (PMC11483899; doi:10.1111/1346-8138.17202)
Supplement: Supplementary file 2 — Data S2. [file JDE-51--s005.pdf]

UCLA SCTC GIT 2.0 Questionnaire

The following questions ask about your digestive (gastrointestinal) symptoms over the past 7 days and how much they have affected your life. For all questions, please choose your answers from the options as indicated. If you are not sure how to answer a question, please select the closest possible answer.

<u>In the past week,</u> please indicate the frequency (number of days) that the following occurred.

	None	1-2 days	3-4 days	5-7 days
Had difficulty swallowing solid foods.	☐	☐	☐	☐
Unpleasant sharp pain or burning sensation in the chest (heartburn)	☐	☐	☐	☐
Felt bitter or sour liquid coming up from the stomach to the mouth (gastric acid reflux)	☐	☐	☐	☐
Heartburn when eating "sour" foods like tomatoes and oranges	☐	☐	☐	☐
Vomited back up (vomited a small amount of food eaten or came up)	☐	☐	☐	☐
Slept with upper body elevated or back upright.	☐	☐	☐	☐
Had nausea/feeling sick	☐	☐	☐	☐
I vomited.	☐	☐	☐	☐

In the past week, please indicate the frequency (number of days) that the following occurred.

	None	1-2 days	3-4 days	5-7 days
I had a feeling of fullness (gas or air in my stomach)	☐	☐	☐	☐
Sometimes the belly would bulge and sometimes it was necessary to loosen belts or unbutton pants and shirts	☐	☐	☐	☐
A little food filled me up.	☐	☐	☐	☐
Lots of farting and gas.	☐	☐	☐	☐
I didn't make it to the bathroom in time and soiled my underwear with stool.	☐	☐	☐	☐
Loose stools.	☐	☐	☐	☐

Over the past week, have you noticed that your stools have become

	No	Yes
Stools were watery.	☐	☐

In the past week, please indicate how often your social life was interrupted by the following (e.g., visiting friends or relatives).

	None	1-2 days	3-4 days	5-7 days
nausea	☐	☐	☐	☐
vomiting	☐	☐	☐	☐
gastralgia	☐	☐	☐	☐
diarrhea	☐	☐	☐	☐
Worries that you might accidentally soil your underwear	☐	☐	☐	☐
sensation of fullness	☐	☐	☐	☐

In the past week, please indicate the frequency (number of days) that the following occurred.

	None	1-2 days	3-4 days	5-7 days
Felt concerned or anxious about gut issues	☐	☐	☐	☐
Embarrassment due to bowel symptoms.	☐	☐	☐	☐
Intestinal symptoms caused sexual problems and problems in the relationship with the partner	☐	☐	☐	☐
I was afraid I might not be able to find a restroom.	☐	☐	☐	☐
Mental depression or weakness due to intestinal symptoms	☐	☐	☐	☐
Avoided or postponed travel due to intestinal symptoms	☐	☐	☐	☐
Anger and irritability due to intestinal symptoms.	☐	☐	☐	☐
Sleep interrupted due to intestinal symptoms.	☐	☐	☐	☐
Felt bowel symptoms worsened due to "stress" or emotional disturbances	☐	☐	☐	☐

<u>Over the past week,</u> have you noticed that your stools have become

	No	Yes
It's getting hard.	☐	☐

<u>In the past week,</u> please indicate how often the following occurred

	None	1-2 days	3-4 days	5-7 days
Constipation or failure to defecate	☐	☐	☐	☐
Stools were hard.	☐	☐	☐	☐
Pain during defecation.	☐	☐	☐	☐
