# Supplementary figures and images for: Reliability, validity, and sensitivity of the Japanese version of the University of California Los Angeles scleroderma clinical trial consortium gastrointestinal tract instrument: Application to efficacy assessment of intravenous immunoglobulin administration
Source: J Dermatol. 2024 Apr 1;51(6):741–51. doi: 10.1111/1346-8138.17202 (PMC11483899; doi:10.1111/1346-8138.17202)

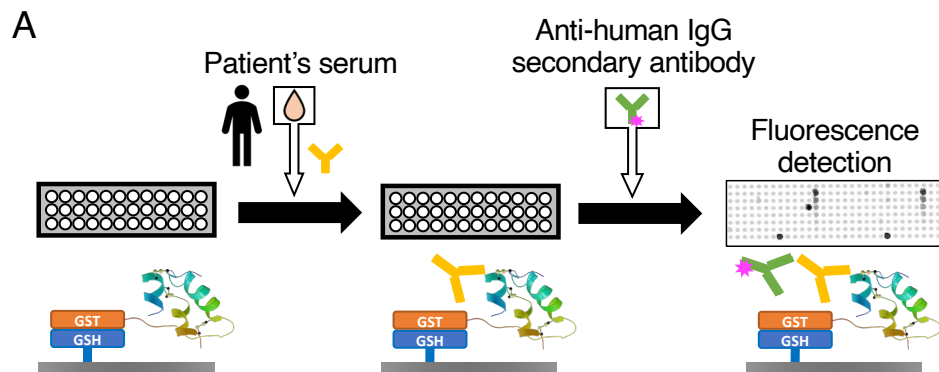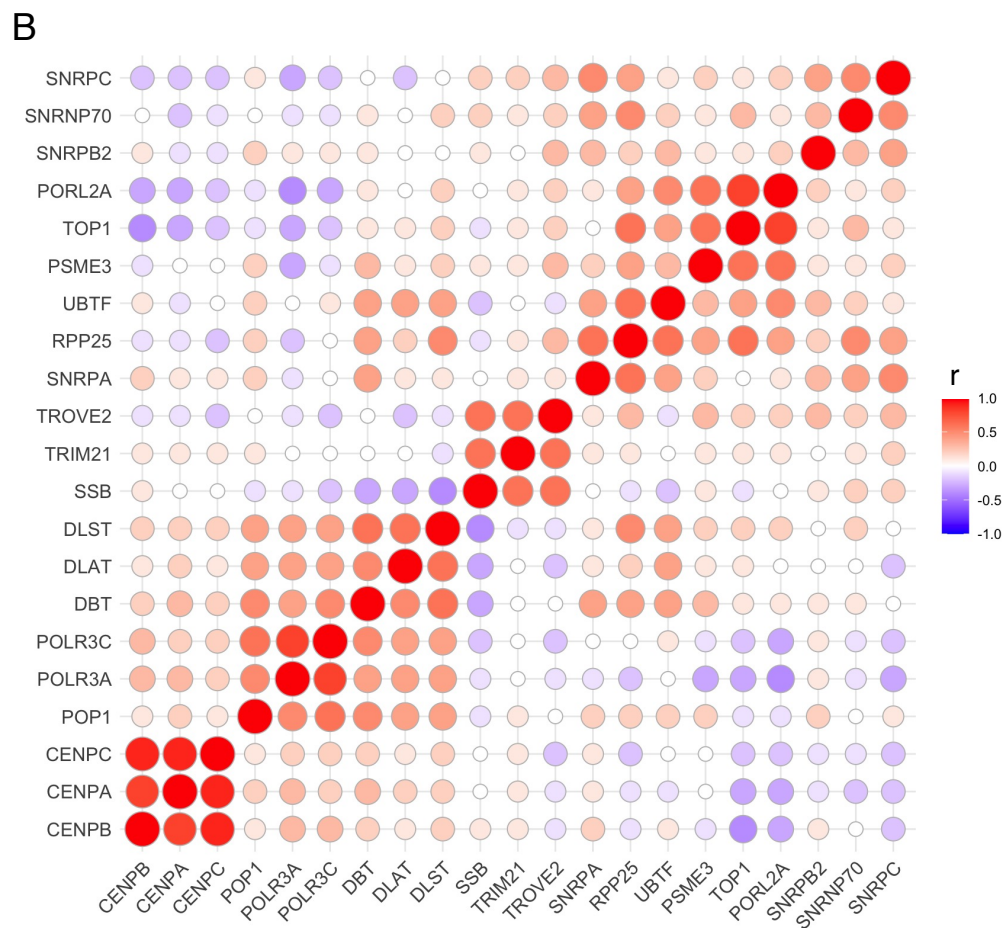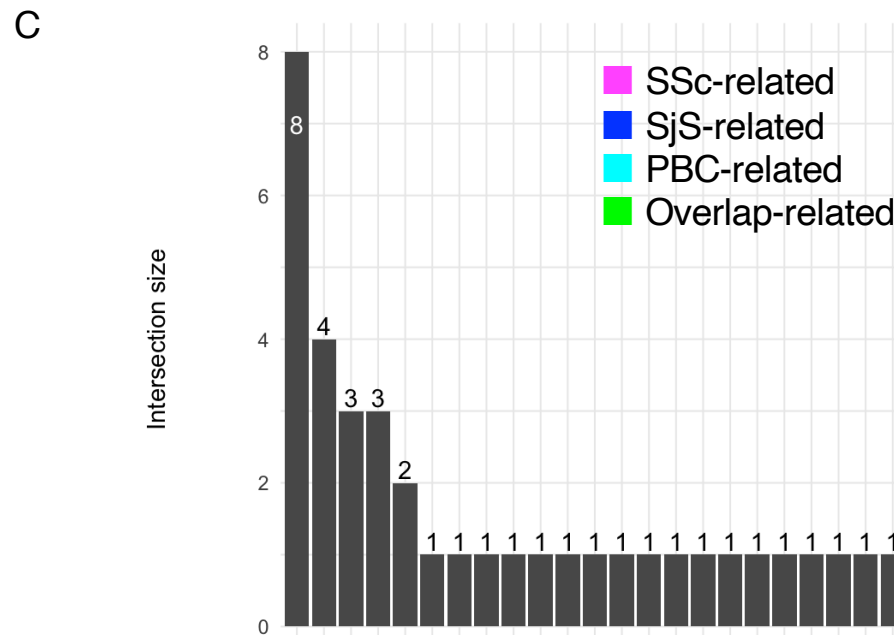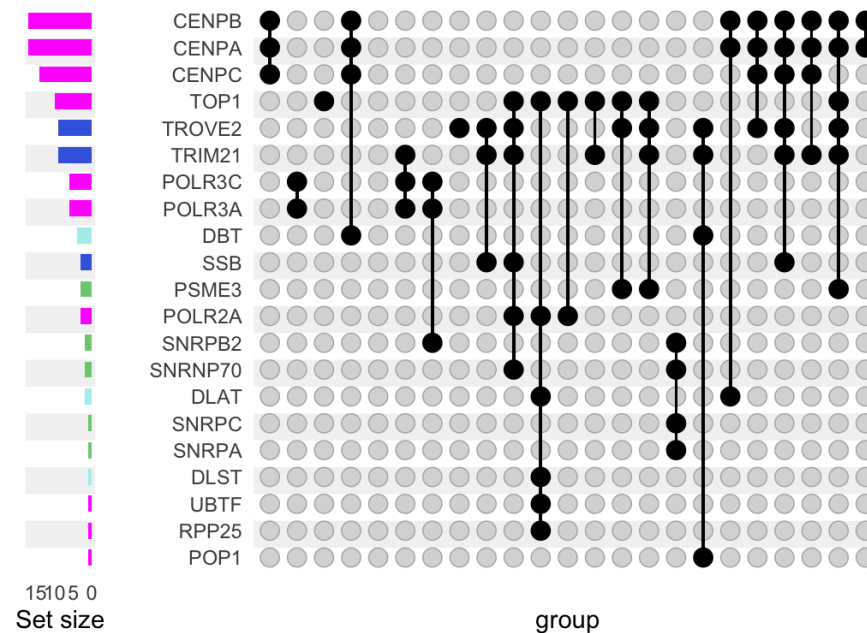

Supplement: Supplementary file 3 — Figure S1. [file JDE-51--s002.pdf]
